# The Role of IGF/IGF-IR-Signaling and Extracellular Matrix Effectors in Bone Sarcoma Pathogenesis

**DOI:** 10.3390/cancers13102478

**Published:** 2021-05-19

**Authors:** George N. Tzanakakis, Eirini-Maria Giatagana, Aikaterini Berdiaki, Ioanna Spyridaki, Kyoko Hida, Monica Neagu, Aristidis M. Tsatsakis, Dragana Nikitovic

**Affiliations:** 1 Laboratory of Histology-Embryology, School of Medicine, University of Crete, 71003 Heraklion, Greece; tzanakak@uoc.gr (G.N.T.); eirini_gt@hotmail.com (E.-M.G.); berdiaki@uoc.gr (A.B.); ispyridaki@uoc.gr (I.S.); 2 Laboratory of Anatomy, School of Medicine, University of Crete, 71003 Heraklion, Greece; 3 Department of Vascular Biology and Molecular Pathology, Hokkaido University Graduate School of Dental Medicine, Sapporo 060-8586, Japan; khida@den.hokudai.ac.jp; 4 Department of Immunology, Victor Babes National Institute of Pathology, 050096 Bucharest, Romania; neagu.monica@gmail.com; 5 Laboratory of Toxicology, School of Medicine, University of Crete, 71003 Heraklion, Greece; tsatsaka@uoc.gr

**Keywords:** bone sarcoma, IGF signaling, IGF-1R, extracellular matrix, tumor microenvironment, cancer therapy, proteoglycans

## Abstract

**Simple Summary:**

Bone sarcomas are mesenchymal origin tumors. Bone sarcoma patients show a variable response or do not respond to chemotherapy. Notably, improving efficient chemotherapy approaches, dealing with chemoresistance, and preventing metastasis pose unmet challenges in sarcoma therapy. Insulin-like growth factors 1 and 2 (IGF-1 and -2) and their respective receptors are a multifactorial system that significantly contributes to bone sarcoma pathogenesis. Most clinical trials aiming at the IGF pathway have had limited success. Developing combinatorial strategies to enhance antitumor responses and better classify the patients that could best benefit from IGF-axis targeting therapies is in order. A plausible approach for developing a combinatorial strategy is to focus on the tumor microenvironment (TME) and processes executed therein. Herewith, we will discuss how the interplay between IGF-signaling and the TME constituents affects bone sarcomas’ basal functions and their response to therapy. Potential direct and adjunct therapeutical implications of the extracellular matrix (ECM) effectors will also be summarized.

**Abstract:**

Bone sarcomas, mesenchymal origin tumors, represent a substantial group of varying neoplasms of a distinct entity. Bone sarcoma patients show a limited response or do not respond to chemotherapy. Notably, developing efficient chemotherapy approaches, dealing with chemoresistance, and preventing metastasis pose unmet challenges in sarcoma therapy. Insulin-like growth factors 1 and 2 (IGF-1 and -2) and their respective receptors are a multifactorial system that significantly contributes to bone sarcoma pathogenesis. Whereas failures have been registered in creating novel targeted therapeutics aiming at the IGF pathway, new agent development should continue, evaluating combinatorial strategies for enhancing antitumor responses and better classifying the patients that could best benefit from these therapies. A plausible approach for developing a combinatorial strategy is to focus on the tumor microenvironment (TME) and processes executed therein. Herewith, we will discuss how the interplay between IGF-signaling and the TME constituents affects sarcomas’ basal functions and their response to therapy. This review highlights key studies focusing on IGF signaling in bone sarcomas, specifically studies underscoring novel properties that make this system an attractive therapeutic target and identifies new relationships that may be exploited. Potential direct and adjunct therapeutical implications of the extracellular matrix (ECM) effectors will also be summarized.

## 1. Introduction

Sarcomas, mesenchymal origin tumors, represent a discrete group of varying neoplasms. Sarcomas develop from transformed mesenchymal cells of various connective tissues, like bone, cartilage, blood or fibrous and adipose tissues, and are broadly defined as bone and soft tissue tumors [1,2], the former being the focus of this review.

Even though bone sarcomas occur in adults, the prevalence of some subtypes is distinctive for the pediatric population [3]. Thus, osteosarcoma and Ewing’s sarcoma (EWS) predominantly present in children and adolescents, whereas chondrosarcoma can present at any age but mainly affects individuals in the 30 to 70 years group [4,5]. These malignancies exhibit heterogeneity at the intertumoral and intratumoral levels partly correlated with their stem cell origin [6]. Indeed, recent studies have provided evidence that osteosarcoma exhibits stem cell-like properties with subpopulations of CD133+ cells, indicating traits of self-renewal, high growth rates, and the formation of spherical colonies [7]. Based on respective tumor molecular bases, histology, or clinical characteristics, bone sarcomas are classified into different subtypes [8,9]. Primary bone tumors are rare malignancies as they account for less than 0.2% of all cancers registered in the EUROCARE (European Cancer Registry-based study on survival and care of cancer patients) database [10]. Bone sarcoma patients show a varying response to chemotherapy [9].

Osteosarcoma is the most common primary bone tumor, with the highest incidence in children and young adults [11,12]. Importantly, conventional osteosarcoma is a high-grade tumor [13]. Even though chemotherapy had initially significantly improved osteosarcoma patients’ prognoses, as chemotherapy treatment of high-grade localized osteosarcoma increases disease-free survival probability from 10–20% to more than 60% [14,15], its effects on survival have plateaued over the last 30 years [16,17]. Notably, improving chemotherapy approaches, dealing with chemoresistance, and preventing metastasis are still major challenges in osteosarcoma therapy [18].

Chondrosarcomas, the second most common bone malignancy, representing 10–20% of all bone malignancies, is the most frequent bone sarcoma of adulthood [19]. Chondrosarcomas are mostly low-grade, locally aggressive, non-metastasizing tumors (grade I-atypical cartilaginous tumors), rather than high-grade (grades II-III), and after wide local excision [19] or after intralesional procedures with curettage and adjuvant treatments usually have a good prognosis [20]. These tumors, however, are resistant to chemotherapy [21]. Likewise, conventional chemotherapy has very limited efficacy in patients with high-grade, advanced chondrosarcoma [22,23], with the highest benefit being noted in mesenchymal and dedifferentiated chondrosarcoma [23]. Likewise, chondrosarcomas are primarily resistant to radiotherapy, except for highly selected cases or palliation [24,25]. Some factors that seem to impair this resistance are the chondrosarcoma extracellular matrix (ECM), the low percentage of dividing cells, and poor vascularity of tumors [26].

The Ewing sarcoma (EWS), an aggressive, primarily pediatric tumor, may develop as a bone sarcoma or a soft-tissue sarcoma [27]. The 2013 WHO classification of sarcomas [28] defines tumors carrying the pathognomonic FET–ETS gene fusions, in which a member of the FET gene family is fused with an ETS transcription factor, with the most common fusion being EWSR1–FLI1, as ‘Ewing sarcoma’ [29]. Notably, the majority of childhood sarcomas, including EWS, exhibit low recurrent genetic alteration except for pathognomonic and uniformly expressed driver mutations [27,30,31]. However, it has recently been suggested that the cooperation of tumorigenic driver-mutations with discrete regulatory germline variants could account for the inter-individual variability of cancer clinical outcomes [32]. EWSR1-FLI1 fusion reprograms the epigenome by introducing de novo enhancers at GGAA microsatellites and modifying the gene regulatory element’s state [33].

Before the development of chemotherapy, just 10% of EWS patients survived, whereas the application of chemotherapy increased survival to 75% in patients with localized tumors. Notably, only 25% of patients with metastatic/recurrent EWS achieve disease regression under current multifunctional treatment options consisting of local control either through surgery or radiation combined with systemic chemotherapy [34,35]. Metastatic patients thus still have a dismal prognosis [35]. Given the limitations of current medical therapies, novel treatment strategies are urgently needed.

Increased expression of IGF-1 and IGF-1R have been reported in the majority of sarcomas, including osteosarcoma [36], EWS [37], and soft tissue sarcomas [38], and has been correlated with disease progression.

## 2. IGF/IGFR-IR Signaling in Cancer

Insulin-like growth factors 1 and 2 (IGF-1 and -2) and their respective receptors are a multifactorial system that regulates metabolism, cell growth, and cancer [39]. Notably, these polypeptide hormones are directly correlated with the growth of both normal and cancer cells. Τhe IGF “family” consists of IGF ligands their cell surface receptors insulin-like growth factor receptor I and II (IGF-IR, IGF-IIR) and the insulin receptor (IR), which execute the biological effects of ligands, as well as six IGF binding proteins (IGFBPs) which fine-tune these effects [40,41].

### 2.1. IGF Receptors

IGF-IR and IGF-IIR have different structures. IGF-IR is a homolog of the insulin receptor (IR), can form functional heterodimers with IR but has a higher affinity to IGF-1/IGF-2 than insulin. Both IGF-IR and IR can bind all three ligands within the family (insulin, IGF-1, and IGF-2) [40,41]. However, IGF-IR appears to be an essential receptor for IGF-1, as it exhibits a significantly higher affinity for IGF-1 than IR. On the other hand, IGF-IIR binds to IGF-2 only and causes its lysosomal degradation [42].

### 2.2. IGF-1 and IGF-2

IGF-I actions are divided into growth hormone (GH)-dependent functions, exerted mainly by hepatic IGF-1 secretion, and those that are not GH dependent and are executed at the level of specific tissues [43]. Epidemiological studies have demonstrated a correlation between high circulating IGF-1 levels and an increased risk of developing cancer [44]. Even though the role of IGF-2 in cancer is controversial, some studies demonstrate a clear correlation of its overexpression with the risk of some cancer development [45].

### 2.3. IGFBs

The free IGF level is modulated by IGF production’s efficacy, serum clearance, and binding affinities to IGFBPs. The majority of circulatory IGFs are bound to one of the six IGFBPs, predominantly IGFBP3 and IGFBP5, which mediate their half-life [46]. The IGFBP levels are higher than those of IGFs and exhibit binding affinities similar to IGF-IR [47]. The majority of IGFBPs antagonize IGFs’activities, whereas IGFBP2 enhances IGF function [48]. IGFBPs also exhibit IGF-independent activities [49], and this function is correlated with cancerogenesis [50].

### 2.4. IGF-IR Downstream Signaling

The activation of IGF-IR initially results in the autophosphorylation of tyrosine 1131, 1135, and 1136 residues and subsequently causes the phosphorylation of transmembrane tyrosines and carboxy terminals [51]. Cytoplasmic “anchor” molecules, including the insulin receptor substrate (IRS) and the Shc-transforming protein 1 (Shc), are recruited by the activated IGF-IR, enhancing growth, differentiation, and protection against apoptosis [52]. The Shc-dependent downstream signaling paths activate mainly RAS-small GTPase (RAS)/proto-oncogene serine/threonine-protein kinase (RAF)/mitogen-activated protein kinase (MAPK). In contrast, the IRS triggers phosphoinositide 3-kinases (PI3K)/Protein kinase B (AKT)/mechanistic target of rapamycin (mTOR) signaling [52].

In recent years, the importance of non-canonical IGF-IR signaling has emerged. In a novel paradigm, IGF-IR is engaged in G-protein coupled receptor (GPCR) signaling, putatively through forming a functional receptor tyrosine kinase (RTK)/GPCR hybrid, which merges the kinase signaling with canonical GPCR characteristics [53]. Therefore, IGF-IR abrogation of the tyrosine kinase-mediated activities may favor IGF-IR/β-arrestin-1/ERK signaling, enhancing tumor resistance mechanisms [54].

Another critical role of IGF-IR in cancer is its involvement in invasion and metastasis through increased β-catenin transcription and E-cadherin cleavage from the actin cytoskeleton. The effect is to disrupt intercellular contacts and facilitate cancer cell mobilization [55,56].

The signaling functions of IGF-IR extend to the nucleus. The endocytosis and translocation of IGF-IR to the nucleus seem to correlate directly with the level of ligand-induced receptor phosphorylation [57,58]. Nuclear IGF-IR binds to enhancer regions and initiates the transcription of target genes as a transcriptional co-activator of lef1/tcf [59]. IGF-IR transcriptional activities lead to increased cyclin D1 and axin2, possibly contributing to IGF-IR promotion of unrestricted cell proliferation and subsequent malignant transformation [59,60].

Moreover, IGF-IR can converge with other signaling pathways. Thus, it can serve as a conduit point for IGF-1/epidermal growth factor (EGF) and estrogen signaling in cancer cell adhesion regulation [61]. Indeed, it has been shown that the crosstalk between estrogen receptors and EGFR/IGF-IR signaling pathways alters cell functions and induces an aberrant expression pattern of matrix molecules in cancer [62].

## 3. IGF-IR/IGF-I Signaling in Sarcoma Pathogenesis

Aberrant expression of IGF pathway members has been determined in various sarcoma types [63]. Even early studies determined an elevated IGF-IR/IGF-I expression in osteosarcoma [64]. Indeed, it has been determined in other cancer models that the overexpression of IGF-IR/IGF-I may be initiated by depressing specific tumor suppressor genes, including BRCA1 and p53 [65,66].

Moreover, IGF-IR was the critical determinant of malignant transformation in EWS required for the EWS/FLI-1 transformation of fibroblasts [67]. Notably, the EWS/FLI-1 fusion gene downregulates the expression of the IGFBP-3 by binding the IGFBP-3 promoter and suppresses its activity. Since IGFBP-3 is a major regulator of IGF-1-dependent proliferation and survival signaling, Prieur et al. showed that the repression of IGFBP-3 is a crucial event in the development of Ewing’s sarcoma [68].

As with other malignancies, sarcoma patients exhibit modified IGFBP circulatory concentrations when compared to healthy subjects. IGF-1, IGF-2, and insulin bind to both types of IGF-IR and IR. Although each receptor has its affinity for these ligands, there are overlapping profiles of action in the target cells, an issue that complicates the mechanisms of their activity [69].

Notably, a generalized IGF-IR signaling input in sarcoma progression was demonstrated by a recent meta-analysis correlating IGF-1R expression with poor outcomes regarding overall survival in sarcoma patients [70]. Likewise, a poor prognosis of patients expressing IGF-I was determined by implementing tissue microarray analysis [71,72].

In the following sections, we will briefly discuss IGF-signaling involvement in the pathogenesis of some bone sarcoma types.

### 3.1. Osteosarcoma

IGF-1 and IGF-1R push osteosarcoma progression through subsequent malignant transformation, proliferation, attenuated susceptibility to apoptosis, and the differentiation of a prone to metastasis phenotype [72,73,74]. Notably, the IGF signaling mediators have now been recognized as biomarkers for primary osteosarcoma detection [75]. A distinct correlation between IGF-IR downstream pathways and osteosarcoma disease progression seems to have been identified. The downstream PI3K/AKT pathway was over-activated during primary osteosarcoma development and pulmonary metastasis, whereas the RAS/MAPK pathway seems to contribute to later stages of pulmonary dissemination [72]. Furthermore, it has been shown that IGFBP5, the most profuse bone IGFBP stored in bone, attenuates tumor growth and human osteosarcoma metastasis [76]. IGF-IR expression has been highly correlated with ABC subfamily G member2 (ABCG2) expression in a cohort of osteosarcoma patients under 10 [77]. As ABCG2 bestows resistance to anticancer drugs [78], these data suggest that the two proteins in combination can be utilized as prognostic factors/therapy determinants.

IGF-1 gene polymorphisms were investigated for the association of risks and outcomes of osteosarcomas. Five single nucleotide polymorphisms (SNPs) of IGF-1 (e.g., SNPs like rs6214, rs6218, rs35767, rs5742612, and rs5742714) were genotyped. Out of all tested SNPs, rs6218 proved to have a predictive role for osteosarcoma’s susceptibility and progression. Moreover, this SNP was associated with a later stage and elevated risk of osteosarcoma metastasis [79]. Furthermore, an exclusive nuclear localization of IGF-1R was associated with progression-free survival and overall survival in osteosarcoma patients treated with IGF-1R Ab therapy [80].

Notably, osteosarcoma, in contrast with other pediatric tumors [30] exhibits a high degree of mutational diversity and copy number variability [75,81]. Indeed, 7–14% of osteosarcoma cases exhibit mutations in IGF signaling genes (IGF1R, IGF1, IGF2R, and IGFBP5). Thus, even taking into account intratumor heterogeneity, these data indicate that taking advantage of anomalies in the osteosarcoma genome could offer novel therapeutic strategies [82].

### 3.2. Chondrosarcoma

IGF-signaling facilitates chondrosarcoma pathogenesis. Thus, treatment of human chondrosarcoma cells with IGFBP3 or IGF inhibitors enhanced their apoptosis rate, whereas mice expressing Gli2 presented fewer tumors upon IGF-2 downregulation. Therefore, Ho et al. suggest that IGF signaling-dependent apoptosis mediates chondrocytes’ malignant transformation [83].

The genetic polymorphisms in IGF-1 pathway members have also been correlated with elevated risk and poor prognosis of conventional chondrosarcoma patients in Chinese populations. Thus, IGF-IR rs2016347 polymorphisms were associated with the risk of lung metastasis of CHS [84].

### 3.3. Ewing’s Sarcoma

Notably, since EWS tumor cells express both IGF-IR and IGF-1, an autocrine loop enhances EWS progression [85]. Moreover, the inhibition of EWS-FLI1 fusion protein decreased IGF-1 and impaired the IGF-1/IGF-1R signaling correlated with increased EWS cell apoptosis, reduced migration, and repressed tumor xenograft growth in a mouse model [86]. Another study focused on EWS cell lines’ high expression of focal adhesion kinase (FAK) transcript and potential interaction with IGF-IR. A dual inhibitor of FAK and IGF-IR, TAE226, was tested along with PF-562,271 as a combination inhibitor of FAK and proline-rich tyrosine kinase 2. TAE226 inhibited the cell growth of various EWS cell lines. The creation of FAK- and IGF-IR- deficient EWS cells induced dysregulation of different signaling pathways. Indeed, TAE226 induced cell cycle arrest, apoptosis, AKT dephosphorylation, and inhibition of invasion. In EWS mouse models, TAE226 was demonstrated to inhibit the local growth of primary tumors and hinder metastasis. Furthermore, the combination of TAE226 and chemotherapy agents showed that TAE226 could exhibit a synergistic effect with conventional chemotherapy and be possibly beneficial for EWS relapse and metastatic patients [87]. Moreover, a recent study demonstrated that CIC-DUX4 Ewing’s sarcoma, an aggressive and often fatal high-grade childhood sarcoma, metastasizes to the lung, utilizing an autocrine IGF-IR/AKT signaling axis [88].

Thus, a deeper understanding of the IGF-signaling molecular facets is obligatory for developing new therapies involving these molecules.

## 4. The Sarcoma Tumor Microenvironment (TME)

The TME consists of tumor cells, non-malignant cells, stromal cells, infiltrating immune cells, and blood vessels embedded in the ECM [89,90]. The tumor cells have evolved mechanisms of interaction with the non-malignant components of the TME, which alter this compartment to facilitate tumor progression [91]. Notably, prominent differences in the immune constituents of the sarcoma TME, e.g., neutrophils, tumor-associated macrophages (TAMs), natural killer (NK) cells, dendritic cells (DCs), and B and T lymphocytes, have been determined and correlated with primary tumor location, sarcoma subtype, genetic or mutational burden and previous therapy exposure [92]. The potentials of the TME have been understudied in sarcoma therapy.

The TME of bone sarcomas is intrinsically different compared to epithelial-derived tumors. Thus, stromal cells are less likely to create distinctive compartments, as usually occurs in epithelial tumors. Indeed, they intermix with tumor cells, immune cells, and other cell types in a tumor-surrounding pseudocapsule. Furthermore, the function of non-malignant cells in sarcoma stroma is well less characterized [93]. Moreover, pediatric and adult sarcomas exhibit distinct characteristics regarding tumor tissue structure [93]. Notably, both tumor and stromal cells produce ECM components that offer structural support and modulate tumor cells’ interaction with the TME.

### The Non-Cellular TME Compartment in Sarcomas

The ECM is a network mainly consisting of collagens, proteoglycans (PGs), glycoproteins, and glycosaminoglycans such as hyaluronic acid (HA). It has the role of a plastic scaffold that bestows physical support to cells within the tissue and regulates the bioactivities of growth factors and cytokines in a time- and location-dependent manner [94]. Aberrant ECM contributes to the stromal cells’ reprogramming and facilitates tumor cell’ growth and dissemination [89,91,95].

In the last couple of decades, the crucial role of the ECM, the non-cellular section of the TME, has been acknowledged in cancer pathogenesis. Previous efforts in classifying the disease and therapy development had focused on the cellular compartment [96]. However, more recent developments have demonstrated the urgent need to understand the ECM component for tumor characterization and efficient therapy development [89,97].

Bone sarcoma extracellular matrices exhibit striking characteristics. Thus, osteosarcoma osteoid is an organic partly mineralized network that mainly consists of type I collagen, glycoproteins, and PGs [98]. The osteoid’s structural components participate in signaling pathways correlated with specific pathogenic phenotypes of bone [99,100]. Indeed, it has been shown that the small leucine-rich proteoglycans (SLRPs), functionally involved in normal bone development and homeostasis [101], mediate various osteosarcoma cell functions [102,103]. Notably, transcriptional analysis of paired normal bone and osteosarcoma samples demonstrated significant alternations regarding mediators of extracellular matrix degradation and collagen biosynthesis [104]. As recently discussed by Cui et al., an increase in the expression of major ECM components, including collagens (I, III, IV and V), fibronectin, laminin, and the PGs (biglycan and lumican), has been determined in osteosarcoma compared to normal bone samples [99]. The HA-binding PG, versican, is likewise overexpressed in osteosarcoma tissues relative to healthy bone tissue and facilitates osteosarcoma cell migration [105]. Considering the ECM as a crucial regulator of tumor progression [100] has allowed the identification of specific molecules of the tumor osteoid as putative therapeutic targets [99].

Chondrosarcoma cells are characterized by intense production of cartilage-like ECM, rich in collagen type II and proteoglycans [26,106,107], with different expression patterns compared to normal tissue [108,109]. Notably, somatic changes of the collagen 2A1 gene were identified in 19.3% of chondrosarcoma and 31.7% of enchondroma tumor cohort cases [110]. Interestingly, a fusion between activin receptor 2A and fibronectin 1 was detected in 57% of synovial chondromatosis cases and in 75% of chondrosarcoma secondary to synovial chondromatosis, showing that fibronectin1 and/or AVCR2A gene rearrangements are present in both benign and malignant synovial chondromatosis, with a higher incidence in malignant disease [111].

Normal chondrocytes predominantly synthesize collagen types II, IX, X, and XI and characteristic proteoglycans, depending on their differentiation state [112]. The fact that the cartilaginous-like matrix production by chondrosarcoma cells is so intensive may indicate they originate from multipotent mesenchymal stem cells, which differentiate along the chondrocytic lineage. Interestingly, despite the malignant transformation, chondrosarcoma cells continue to express some molecules that characterize normal tissue [112,113,114].

Regarding radiotherapy and conventional chemotherapy, chondrosarcoma is characterized as a resistant lesion [115] due to the tumor’s specific hallmarks. Chondrosarcoma tumor tissue, like hyaline, is characterized by a dense ECM with poor blood and lymph vascularity, on which a low percentage of dividing cells is embedded. Thus, the ECM forms a physical semi-permeable barrier, inhibiting cytotoxic agents reaching their target, i.e., chondrosarcoma cells, while reduced blood circulation creates severe chronichypoxia [116]. Moreover, the Schwan chondrosarcoma ECM disturbance by modifying the synthesis of ECM components, mainly PGs, attenuates this tumor growth [117]. The participation of non-cellular TEM components required during sarcoma progression and their interaction with IGF-effectors has not been systematically investigated.

## 5. Interplay between Matrix Effectors and IGF/IGF-IR Signaling Regulates Sarcoma Functions

Matrix molecules participate in different signaling pathways and finally control cellular behavior [118]. Their synergistic action with IGF/IGF-IR signaling pathway is involved in cancer’s pathogenesis [119,120,121,122]. The interactions are perpetrated in two directions: (i) IGF/IGF-IR regulates sarcoma matrix effector synthesis, structure/organization, and downstream functions; and (ii) sarcoma matrix effectors modulate IGF/IGF-IR pathway restricted signaling.

### 5.1. IGF/IGF-IR Regulate Sarcoma Matrix Effectors Synthesis, Structure/Organization, and Downstream Functions

#### 5.1.1. Proteoglycans

The ECM of chondrosarcoma and osteosarcoma has a high PG content, which contributes significantly to the network’s physicochemical characteristics [98,123]. PGs are hybrid molecules composed of protein core into which one or more GAG chains, e.g., heparan sulfate (HS), chondroitin sulfate/dermatan sulfate (CS/DS), or keratan sulfate (KS) are bound [124]. Based on their cellular and subcellular deposition, PGs are classified into the cell membrane, pericellular extracellular, and intracellular categories [124]. Even early reports have shown that IGF-1 and IGF-2 maintain, in an autocrine manner, the high PG synthesis in in vitro chondrosarcoma models [125]. Indeed, IGF-I and IGF-II enhance aggrecan expression, considered a typical differentiation marker of chondrocytes [125]. Aggrecan, which is classified as a hyalectan, extracellular PG, can regulate vital cellular functions and contribute significantly to the pericellular matrix organization [126]. The hyalectan members are subjected to alternative splicing and exhibit variable glycosylation patterns, allowing them to discretely link cell surfaces with the ECM networks [107]. Hyalectans have the ability to aggregate, creating supramolecular complexes, which in the case of aggrecan constitute the key load-bearing cartilage component [126].

Notably, change in the pattern of alternative aggrecan mRNA splicing is associated with malignant transformation of chondrocytes [127]. IGF-1 enhances both PG and p21 expression of SW1353 chondrosarcoma cells in a manner correlated with chondrosarcoma differentiation [128]. Interestingly, aggrecan expression increases when human chondrosarcoma HCS-2/8 cells are cultured during extended periods in a 5% low-oxygen atmosphere. Since hypoxia is strongly evident in chondrosarcoma, upregulated aggrecan expression is suggested to be a protective factor for chondrosarcoma cell survival [129].

The IGF/IGFBP was shown to be a convergence point in the regulation of aggrecan synthesis. When costal embryonic rat chondrocytes were treated with parathyroid hormone (PTH) in a dose-dependent manner, a significant increase in aggrecan synthesis was observed. A neutralizing IGF-I resulted in the inhibition of PTH-stimulated aggrecan mRNA synthesis, whereas the addition of a neutralizing antibody to IGFBP-2 resulted in increased synthesis. These results show that PTH affects aggrecan synthesis through an IGF/IGFBP axis and indicate that the IGF-1 local increase may increase cartilage type PG synthesis and maintenance of the cartilage phenotype [130].

Aggrecan is retained at the chondrosarcoma cell membrane via its binding to cell-associated HA [131]. Significantly, HA deposition is enhanced to the chondrosarcoma peritumoral stroma and tumor tissue compared to healthy tissue levels. On the other hand, HA synthase levels are downregulated in the tumor tissues suggesting modulation in HA synthesis and turnover. Moreover, the pericellular matrix changes are likely associated with chemotherapy resistance [132]. Therefore, modulating HA content and the resulting PG aggregation in the pericellular matrix, in combination with chemotherapeutic agents, could increase the efficiency of therapy.

PGs are being tested in preclinical trials as chondrosarcoma therapy targets [133]. Notably, new treatment options are urgently required in chondrosarcoma, particularly chondrosarcomas with a well-differentiated hyaline cartilage-like ECM (e.g., collagen II and proteoglycan-rich), notoriously resistant to drug penetration, and with the potential for progression towards a higher grade [134].

Interestingly, a correlation between IGF-1 signaling and PG synthesis was identified in osteosarcoma. Thus, the xylosyltransferases I and II (XT I and II), responsible for initiating the PG-glycosylation process, are the rate-limiting step in PG biosynthesis [135]. IGF-1 treatment was shown to stimulate XTI and alkaline phosphatase expression in Saos 2 cells indicating its crucial role in osteosarcoma cell PG synthesis [135].

Osteosarcoma PG content seems to strongly affect these cancer cells’ interactions with the microenvironment. Thus, the expression of the cell membrane HSPG syndecan-4 and its matrix binding partner, fibronectin, were correlated with distant metastasis and shorter overall survival in a cohort of osteosarcoma patients [136]. Notably, targets of microRNA-199a-3p (miR-199a-3p) in osteosarcoma have been found to be enriched in PG genes, demonstrating their role in supporting osteosarcoma oncogenic potential [137].

#### 5.1.2. Collagens

Collagen fibrils are the major mechanical component of the ECM [138]. They are present in different connective tissues, including cartilage and bones [139]. Collagens participate in the mechanical resistance and resilience of connective tissues and act as signaling molecules to arrange cellular shape and behavior. These fibril components of ECM communicate with cells by three types of cell surface receptors, integrins, discoidin domain receptors, and glycoprotein VI, and finally trigger a variety of signaling pathways upon collagen-binding [140].

Early studies demonstrated that IGF-1 stimulates osteosarcoma cell collagen I production and that this effect was attenuated through the action of the insulin-like growth factor-binding protein-4 (IGFBP4) in a concentration-dependent manner [141]. IGF-1 also regulates the osteosarcoma collagen matrix by decreasing cysteine protease activities [142]. Moreover, IGF-1 and/or IGFBP-5 participate in the estrogen-mediated regulation of PTH action on Saos2 osteosarcoma cell proliferation and collagen I production [143].

There is strong evidence that collagen I is closely related to bone diseases, bone cancers, and cancer-related bone metastases [144,145], and the above mechanism possibly describes the synergistic role of collagen I and IGF-1 to promote osteoblastic lineage cell growth.

Collagen II is the primary collagen in cartilage [146]. Zhang et al. have shown that IGF-1 upregulates Collagen II expression at both the mRNA and protein level in rat endplate chondrocytes isolated from the cervical spine. Furthermore, IGF-1- induced collagen IIa1 gene expression requires de novo mRNA transcription and de novo protein synthesis. IGF-1 action is mediated by PI3K/Akt signaling pathway, as chemical inhibition of PI3K and, therefore, deactivation of Akt abolishes the IGF-1 induced COLII upregulation [147]. Even if very little is known about type II collagen and its relation to cancer, some studies indicate that this type of collagen has anti-oncogenic properties in bone tumors as it inhibits osteoclast survival and induces tumor cell death [148,149]. Interestingly, collagen II is frequently mutated in chondrosarcoma [150].

IGF-1/IGF-IR signaling pathway seems to interact with collagen receptors to control collagen biosynthesis and cellular functions. It is reported that in human fibroblasts, collagen biosynthesis is regulated by both IGF-IR and β1-integrin receptors through proteins participating in pathways generated by these receptors, such as PI3K, ERK 1/2, Akt, and mTOR [151].

Cancer cells and tumor-associated macrophages also produce collagen, and its aberrant biosynthesis can be regulated through mutated genes, transcription factors, signaling pathways, and receptors [152].

#### 5.1.3. Adhesion Molecules

Chao et al.’s very recent study demonstrates an interplay between the IGF signaling pathway and the adhesion molecule vascular cell adhesion protein 1 (VCAM-1) in osteosarcoma cells [153]. IGFBP-3, an essential regulator of IGF-1 signaling [154,155], was shown to facilitate VCAM-1 expression that promoted human osteosarcoma cell migration capacity through the PI3K, Akt, and AP-1 signaling pathways [153]. IGF-1 has also been correlated with a5b1 integrin-dependent human chondrosarcoma metastasis. Indeed, IGF-1 modulated a5b1 integrin expression via the IGF-IR, PI3K, Akt, IKKa/b, and NF-kB-dependent pathway causing an increase in migration of human chondrosarcoma cells posing as an effective therapy tool [156].

#### 5.1.4. Proteases

A therapeutic potential was also found for IGFBP-3 in EWS. Forced expression of IGFBP-3 in TC-71 Ewing sarcoma cells induced decreased production and/or activity of matrix metalloprotease-9 (MMP-9) and vascular endothelial factor (VEGF)-A, that abolished EWS metastatic ability [157]. Furthermore, IGF-IR was differentially expressed in human sarcomas, and the targeted blockade of the IGF-IR pathway inhibited human osteosarcoma migration through downregulation of MMP-2 and -9 expression [158].

#### 5.1.5. Summary

The above studies clearly show that the IGF-axis affects the ECM components’ expression/activity to facilitate sarcoma progression (Table 1). Changes in the ECM enhance hallmarks of cancer, including tumor growth, survival, and metastasis. Thus, IGF signaling has a vital role in the cancerization of the sarcoma microenvironment.

### 5.2. Matrix Effectors Modulate IGF/IGF-IR Pathway Restricted Signaling

The organization of the ECM network structure modulates IGF-IR signaling. Thus, in vitro 3D environments enhance the canonical IGF-IR signal cascade’s attenuation through mechanistic target of rapamycin (mTOR). Notably, 3D environments facilitated a decrease in the clathrin-dependent nuclear localization and transcriptional activity of IGF-IR [159]. Therefore, modulating the matrix network could contribute to cancer therapies directed at the IGF-signaling pathway.

Along these lines, it has been shown that heparin affects the IGF-1/IGF-2-dependent binding of IGFBP-2 to the ECM of the malignant osteoblastic cells [160]. These data agree with the notion that upon IGF-1/IGF-2 binding to IGFBP2, the resulting complex attaches to the HSPGs component of the ECM [161]. This mechanism supports osteoblast growth and offers protection against apoptosis [161]. Notably, aberrant expression of insulin-like growth factor 2 mRNA binding protein 3 (IGF2BP3) is correlated with osteosarcoma’s metastasis to the lungs [162].

Sarcomas and their mesenchymal precursor cells express the cell membrane chondroitin sulfate proteoglycan 4 (NG2/CSPG4) [163]. Hsu et al. showed that NG2/CSPG4 expression is positively correlated with cell proliferation and negatively to apoptosis in established sarcomas. Gene deletion of this PG or NG2/CSPG4 directed immunotherapy affects tumor behavior depending on the developmental stage. Thus, upon NG2/CSPG4 downregulation in established tumors in murine and human sarcoma models, increased caspase 7 and IGFBP3 genes’ expression reduces tumor size. On the other hand, deletion of NG2/CSPG4 at tumor initiation activates IGF signaling, a pathway known to positively regulate soft-tissue sarcoma growth. These data suggest that targeting NG2/CSPG4 and its effects on IGF-signaling is a potential, tumor stage-dependent, therapeutic approach [164].

Many studies have implicated the participation of cell surface HSPGs, such as glypicans and syndecans, in cancer progression and metastasis. Aberrant expression of glypicans is correlated with distinct pediatric embryonal tumors’ pathogenesis [165]. Loss-of-function mutations of the glypican-3 (GPC-3) gene are the cause of the human Simpson-Golabi-Behmel syndrome [166] an X-linked overgrowth disorder with a predisposition to GPC3-expressing cancers [165]. Indeed, GPC-3 binding to growth factors such as IGF-2 in different tumor cell types affects these cells’ survival, as GPC3 can induce apoptosis or inhibit proliferation in a cell line-specific manner, and these cells can be rescued by IGF-2 signaling [167]. Moreover, syndecan 2 is a cell surface HSPG, with emerging participation in mesenchymal and epithelial tumor pathogenesis [168].

SLRPs, classified as extracellular PGs, comprise the largest class of PGs. The role of SLRPs in sarcoma progression is well established [89,100,102,169]. SLRPs can bind with various biologic mediators, including growth factors, to modulate signaling pathways that participate in regulating basal cellular functions, like proliferation, migration, and differentiation correlated both to homeostasis and pathological conditions [102,103,169].

The class I SLRP, decorin, is a macromolecule with anti-oncogenic action [170]. Decorin is well established to modulate IGF-IR signaling in tumorigenesis. Thus, in cancer cells, decorin was shown to attenuate ligand-dependent IGF-IR activation and downstream signaling in a dose-dependent manner [171]. Additionally, prolonged exposure to decorin did not affect IGF-IR stability, with or without IGF-1 stimulation. On the other hand, downregulation of IGF-IR induces a switch resulting in enhanced IGF-2/IR-A signaling in cancer. This mechanism has been verified in IGF-IR-deficient osteoblasts [172]. Thus, IGF-IR treatments based on decorin that attenuate but do not abolish IGF-IR signaling are potential therapeutic approaches to prevent IGF-IR-dependent chemoresistance. Interestingly, a recent study demonstrated that decorin-coated titanium substrates abolished the oncogenic potential of osteosarcoma cells but, on the other hand, stimulated the proliferation of pre-osteoblasts [173], suggesting that decorin exerts specific antitumor action.

We recently showed that biglycan enhances both basal and IGF-1-dependent osteosarcoma cell proliferation. These effects were mediated through the IGF-IR receptor, whose activation is strongly attenuated in biglycan-deficient MG63 osteosarcoma cells. In parallel, the down-regulation of biglycan significantly inhibits both basal and IGF-1 induced Erk1/2 activation, an essential downstream mediator of IGF-1 signaling. An interaction between β-catenin and the activated IGF-IR, which is increased upon treating MG63 cells with exogenous biglycan, was determined. Biglycan, thus, through a wnt/β-catenin/IGF-IR signaling axis, enhances osteosarcoma cell growth [120]. Notably, Wnt-5a expression was correlated with disease severity in osteosarcoma [174]. As IGF/IGF-IR also enhances biglycan expression in MG63 cells, this mechanism could plausibly be a vicious loop supporting osteosarcoma cell growth and contributing to the initiation of IGF-correlated chemotherapy resistance.

**Figure 1:**
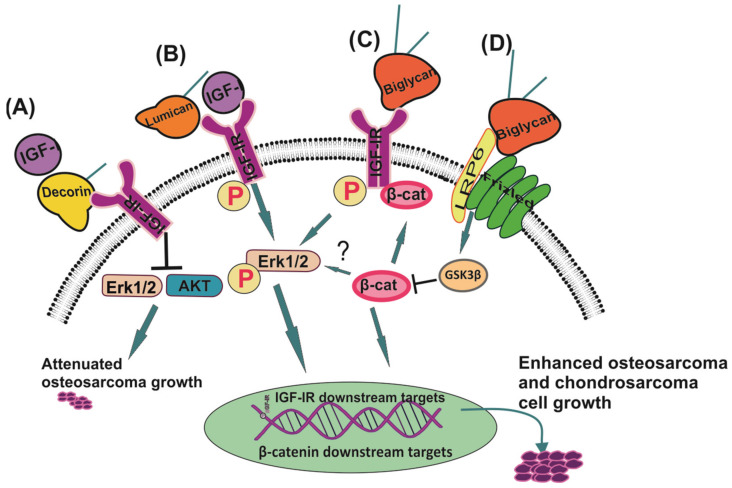
The role of SLRPs on IGF/IGF-IR-dependent cell growth. (**A**) Decorin attenuates ligand-dependent IGF-IR activation and inhibits IGF-IR-restricted Erk1/2 and AKT signaling correlated with decreased osteosarcoma oncogenic potential. (**B**) Lumican forms a complex with IGF-IR to stimulate basal and IGF-1-induced chondrosarcoma cell growth in an ERK-dependent manner. (**C**) Biglycan co-localizes with IGF-IR to enhance its basal and IGF-1-dependent activation and correlated osteosarcoma cell growth. (**D**) Biglycan binds to LRP6/frizled complex initiating a convergence of β-catenin/IGF-IR signaling facilitating osteosarcoma cell growth.

Lumican, a class II SLRP, can likewise interact with the IGF-1/IGF-IR signaling pathway to regulate sarcoma growth. Lumican expressed and secreted by HTB94 human chondrosarcoma cells enhances these cells’ proliferation. On the other hand, lumican deficiency significantly inhibits basal and IGF-1-induced HTB94 cell growth. Moreover, the phosphorylation levels of IGF-IR are strongly attenuated in lumican-deficient cells. Furthermore, lumican levels affect ERK1/2 activation, which seems crucial to IGF-1-dependent HTB94 cell growth [175]. The interaction of IGF-IR and SLRPs in the regulation of sarcoma cell functions is depicted in Figure 1.

IGF action is known to be modulated by proteases through cleavage of inhibitory IGF-binding proteins, resulting in altered cell proliferation, migration, and survival. The role of PAPP-A (pregnancy-associated plasma protein-A), a zinc metalloproteinase, has been studied in several cancers [176]. PAPP-A is one of the top five membrane-associated proteins overexpressed in Ewing sarcoma and, thus, a potentially targetable cell surface antigen [177,178]. The knocking out of the PAPP-A gene in EWS cells diminished free IGF-I, decreased cell growth, and downregulated pathways associated with disrupted IGF signaling [177]. Table 2 presents examples of matrix regulators that affect the IGF-axis and contribute to sarcoma progression.

## 6. IGF/IGF-IR Signaling Regulates Tumor Immune Response—Potential Therapeutic Application in Sarcomas

Notably, the solid tumor microenvironment exhibits characteristics that attenuate efficient antitumor immune response [180,181]. Metabolic deregulations are associated with carcinogenesis, e.g., increased serum insulin levels and free IGF-1 favor cell proliferation and affect the immune response pushing the cellular microenvironment towards carcinogenesis [182,183,184,185]. Thus, there is a clear relationship between the immune system and IGF-signaling, which can govern immune responses’ quality and amplitude [186].

A novel emerging immunotherapy for sarcomas is the chimeric antigen receptor (CAR)-T cell therapy. CAR-T cell therapeutic approach fuses a specific antibody-derived single-chain variable fragment (scFv) onto a T-cell able to recognize a specific tumor-associated antigen and release effector function upon binding to the antigen [187]. IGF-IR is one of the sarcoma-associated antigens suitable for CAR-T cell treatment. Thus, IGF1R and tyrosine kinase-like orphan receptor 1 (ROR1) CAR-T cells obtained from sarcoma patients could secrete Inf-γ and pronouncedly attenuate tumor growth in systemically disseminated and localized osteosarcoma xenograft mouse models [188]. Another significant challenge of applying CAR-T cell therapy is circumventing the osteosarcoma microenvironment’s immune evasion [189].

Thus, myeloid-derived suppressor cells (MDSCs) are a subset of immature monocytic and granulocytic cells that inhibit immune responses via various mechanisms such as activation of regulatory T cells (Treg) and oxidative stress and nutrient depletion. TME is enriched with MDSCs in parallel with the increase of the tumor burden [190]. MDSCs block the targeting of various sarcoma types by chimeric antigen receptor (CAR)-T cells. Therefore, adequate co-treatment is needed to improve CAR-T cells’ efficacy for sarcomas [191].

Likewise, IGFBP-6, shown to be involved in tumorigenesis by promoting cancer cell migration [192], contributes to many immune-related processes, such as pro-B-cell development in vitro and chemotaxis induction of monocytes and T-cells [193].

Due to the EWS’ slow rate of mutations and few neo-antigens various proteomic/genomic studies were initiated. Recently, EWS surfaceome analysis showed exciting results regarding the possible future immunotherapeutics. Pregnancy-associated plasma protein-A (PAPP-A) promotes fetal growth by inducing IGF-signaling. The study of EWS surfaceome identified 11 highly differentially overexpressed genes, out of which PAPP-A has an important differential expression. The utilization of PAPP-A knockout and anti-PAPP-A antibodies treatment in EWS cell lines demonstrated the IGF-1 involvement in cellular survival [177]. Moreover, treated as mentioned, EWS cells exhibited a diminished growth in orthotopic xenografts. The PAPP-A gene knockout induced interferon (IFN)-response genes and enhanced the antigen processing/presentation pathway. Thus, this recent study showed that the EWS surfaceome contains essential molecules that can be therapeutic targets. Among these, PAPP-A stands out as a novel link between IGF-1 signaling and immune evasion in cancer [177].

Another approach for EWS is treating activated NK cells with IGF-IR-specific antibodies. Treated NK cells exhibit enhanced expansion. Therefore, a combination of adaptive NK cell transfer with IGF-IR targeting may be an option to eliminate minimal residual disease [194].

One can imagine potential future avenues in combinatorial therapies, namely the design of approaches targeting the collagen components in the ECM, attenuating the barrier properties of the tumor’s pseudocapsule, facilitating antitumor agents penetrability, and access to the tumor bed. Indeed, CAR-T cell therapy in solid tumors does not show the positive results reported for hematological malignancies because the TME-dependent factors undermine an effective antitumor immune response [180].

## 7. IGF Signaling in Tumor Angiogenesis

Tumor blood vessels are an essential component of the TME and critically enhance tumor growth and metastasis by providing oxygen and nutrients to tumor cells. Most tumors may become dormant without angiogenesis at a diameter of 2–3 mm [195]. Thus, tumor endothelial cells (TECs) have been an important therapeutic target for anticancer strategy. Currently, most angiogenesis inhibitors block vascular endothelial growth factor (VEGF) signaling. Angiogenic inhibitors such as bevacizumab, a humanized anti-VEGF antibody [196], have been used widely in clinics combined with chemotherapeutic drugs or immune checkpoint inhibitors. VEGF is established as a permeability factor [179]; thus, angiogenic inhibitors suppress the growth of tumors and normalize immature blood vessel structures and improve the delivery of oxygen and drugs. Furthermore, infiltration of immune cells such as cytotoxic T cells increases and results in the facilitation of immune checkpoint inhibitors (ICI) effects. This is why angiogenic inhibitors are combined with ICI as a current therapeutic strategy in several cancers.

TECs cover the inner surfaces of tumor blood vessels. Several reports have demonstrated that TECs are abnormal, and their abnormality is one of the causes of resistance to antiangiogenic therapy. TECs exhibit a higher VEGF receptor expression level and express angiogenic growth factors, such as VEGF. Thus, anti-VEGF drugs target not only tumor cell-secreting factors, and may target TECs directly [197].

However, anti-VEGF drugs can be correlated with various complications such as hypertension, hand-foot syndrome, proteinuria, and thyroid dysfunction due to the key role of VEGF for normal blood vessel homeostasis [198]. Additionally, drug resistance may occur, as long-term antiangiogenic therapy leads to tumor hypoxia and induces tumor aggressive behavior [199].

Additionally, TECs show heterogeneity communicating with the surrounding TME. TEC isolated from high metastatic tumors showed proangiogenic phenotype, drug resistance, and chromosomal abnormality, unlike TECs isolated from low metastatic tumors [200]. Microenvironmental factors may alter TEC phenotype. Hypoxia causes chromosome abnormality and excess VEGF, and inflammatory cytokines secretion or low pH induce aberrant TEC phenotype [201,202,203].

Since resistance to anti-VEGF therapy has been determined, the complementary input and the increase of other angiogenic factor expressions have been proposed [204]. Thus, besides VEGF, bFGF, PDGF, and angiopoietin have been considered to be angiogenic factors. IGF-1 also stimulates angiogenesis via activating PI3K/Akt pathway [205].

IGF-IR has been suggested as a potential convergence point in regulating angiogenesis. Indeed, IGF/IGF-1R axis enhances angiogenesis as IGFBP-3 and -5 that neutralize the effects of IGFs downregulate the angiogenic process [206]. This could be an important point, as enhanced IGF-IR signaling, due to strong downregulation of IGFBP-3, has been determined as obligatory in EWS development.

Notably, targeting IGF-1R-with figitumumab (CP751871) resulted in attenuated IGF-1R signaling correlated with a significant downregulation of VEGF in several sarcoma xenografts, including osteosarcoma and EWS [207]. Therefore, IGF-IR in these in vivo models regulates VEGF secretion and activity. Notably, treatment with rapamycin did not downregulate VEGF in tumors and exhibited synergistic action only in tumor cells where VEGF was strongly suppressed by figitumumab [207]. Thus, it has been elucidated that tumor angiogenesis is regulated in a more complex manner than considered before.

The ECM network is extensively involved in angiogenesis and is obligatory in maintaining vascular homeostasis [208]. The PG ECM component contributes significantly to angiogenesis, as it regulates the bioavailability of HS-binding growth factors such as VEGF or FGF [209,210].

The ECM surrounding TEC is altered and, among other things, contains enhanced levels of matrix proteins, like collagens [203,211]. Additionally, we have reported that TECs express a higher level of the SLRP, biglycan, compared to normal endothelial cells. Biglycan secreted from TECs enhances proangiogenic phenotype in TECs in an autocrine manner. Furthermore, TECs attract tumor cells by secreting biglycan, which induces intravasation, followed by distant metastasis [212]. TEC-biglycan levels correlate with lower relapse-free survival or overall survival in lung cancer patients [213]. Moreover, in the osteosarcoma model, we have identified an IGF-IR/biglycan loop [120], which regulates these cells’ growth, suggesting possible critical interaction between the ECM and IGF-IR in the modulation of both tumor growth and angiogenesis.

Furthermore, recent studies have revealed that IGF-1 increases biglycan protein translation by preventing ADAMTS5-mediated degradation indicating a new role of IGF-1 regulating biglycan expression [214]. Since biglycan activates fibroblasts, inducing tumor stiffness [215] and facilitating tumor cell invasion, its interactions with IGF-signaling could present a plausible cancer therapy target.

Taken together, tumor angiogenesis is regulated by IGF, not only by VEGF. Tumor matrix including collagen and proteoglycans are also regulators of tumor angiogenesis.

## 8. Data from Clinical Trials Focusing on Targeting the IGF-IR

Drug development programs, focusing on targeting the IGF-IR, span ten years. Figi-tumumab, a monoclonal antibody against IGF-IR, was developed in 2011 for the treatment of various types of cancer, e.g., adrenocortical carcinoma [216] non-small cell lung cancer (NSCLC) [217], but further development was terminated due to severe adverse effects [218].

**Figure 2:**
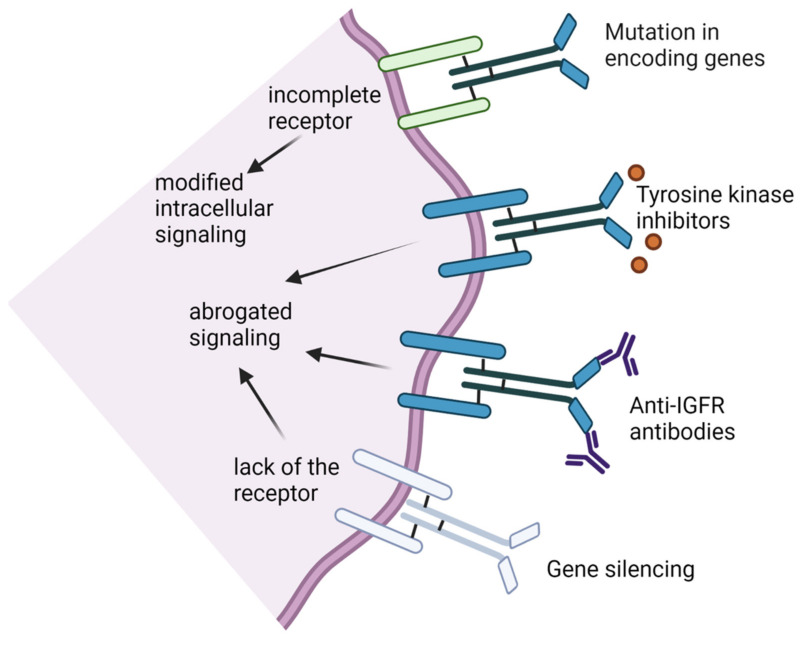
Various therapeutic approaches to targeting IGF-1R receptors. Mutation inducers in the gene that encodes the receptor result in proteins that lack beta-subunits (dominant-negative receptors); tyrosine kinase (TK) inhibitors that hinder intracellular signaling; anti-IGF-IR antibodies which inactivate receptor functionality; gene silencing that blocks protein expression in the transcription or translation phase.

To date, the main approaches for targeting the IGF-IR receptors (Figure 2) involve (i) inhibition of tyrosine kinase (TK), (ii) abrogation of downstream intracellular signaling, (iii) inactivation of inactivating receptor functionality, (iv) induction of mutation in the gene that encodes the receptor leading to proteins that lack beta-subunits and (v) gene silencing that blocks protein expression in the transcription or translation phase [41].

IGF/IGF-IR signaling has, to date in sarcomas, been best studied in EWS. EWS has one of the lowest somatic mutation rates in cancer; thus, there are scarce druggable mutations and/or neoantigens. Several EWS drugs targeting the IGF axis have reached the clinical stage [219].

The IGF-1R pathway deregulation in EWS can be the result of the EWSR1-FLI1 translocation. Indeed, clinical trials have demonstrated that the utilization of IGF-1R inhibitor as a single agent or combination led to favorable clinical responses: 0.7% complete responses, 11% partial and durable responses, and 21% stable diseases. The authors point out that the IGF-IR pathway should be further explored, and new biomarkers identified for selecting patients that might best benefit from treatment [220].

A cohort of 47 pediatric patients with refractory solid tumors (76% were EWS patients and 24% various sarcoma patients) was treated with Cixutumumab in a phase I/II study to determine the recommended phase II dose and to evaluate anticancer activity in EWS. This monoclonal antibody against the IGF-IR exhibits a high affinity for IGF-IR, attenuates its cell-surface expression, and abrogates interactions with IGF-1/IGF-II ligands. Cixutumumab was well tolerated in these pediatric patients but had limited single-agent activity In EWS patients [221]. Furthermore, Cixutumumab was well tolerated in a phase II study but induced limited objective single-agent activity, whereas 15% of patients exhibited prolonged stable disease [222]. Nonetheless, in a subset of EWS patients, a strong dependence of disease progression on IGF-IR activity was determined. Thus, a complete response was determined in a few EWS patients; the partial response was achieved by 2–12% of patients, whereas disease stabilization was determined in 16% to 40% of patients [223,224,225,226].

Importantly, patients with lower pretreatment levels of circulating free IGF -1 exhibited higher overall survival [224]. Moreover, Anderson et al. report that a phase II study where osteosarcoma and EWS metastatic sarcoma patients were treated with Robatumumab identified 6/84 patients who achieved remission and remained healthy after continuous Robatumumab treatment for a period longer than four years [227]. A summary of clinical trials where the anti-IGF-IR mAbs target the IGF axis in EWS and other sarcoma types is presented in Table 3.

These data highlight the need to define markers that identify subsets of patients capable of positive response. Indeed, the expression of IGF-signaling mediators in EWS patients can be used for differentiating between subtypes of patients. This stratification can prognosticate distinct outcomes and personalized treatment protocol. A cohort of 290 EWS biopsies was analyzed for IGF-IR, IR, IGF-1, and specific intracellular mediators (e.g., IRS1, p-ERK) expressions. Notably, IGF-IR and/or IR were expressed in all EWS tumor samples. In this cohort, IGF-IR, IR, and IGF-1 high mRNA expression were significantly associated with the patients’ more favorable clinical outcomes.

Moreover, higher circulating levels of IGF-I were found to be correlated with a lower risk of disease progression and death [229]. These clinical data contrast with the definition that higher expressions of IGF/IGF-IR are associated with higher aggressiveness and indicate that in some stages of cancer development, the transformation malignancy and unsatisfactory therapy response can be correlated with downregulation of IGF signaling. The obtained data highlight the extreme complexity of this axis input in sarcoma pathogenesis.

The utmiR-939 exerted tumor-suppressing roles in osteosarcoma cells’ aggressivity by directly targeting IGF-IR and inactivating the PI3K/AKT pathway [230]. The initial preclinical data were promising; however, the thus-far implemented phase I/II clinical trials on the use of IGF-IR inhibitors in osteosarcoma have given unsatisfactory results [227,228].

Therefore, one can characterize IGF-IR as one of the co-drivers of osteosarcoma progression, even though its sole inhibition is not a clinical option [231]. An alternative would be treating a subset of patients who overexpress IGF-IR with IGF-IR inhibitors as an innovative treatment approach [232]. The other option would be to identify IGF-IR signaling partners and abrogate their interactions.

Another point to be taken into consideration is the identification of mutations in IGF signaling genes. In a recent study of 112 adult and pediatric osteosarcoma cases, these mutations were detected in 7%, whereas in an additional 87 osteosarcomas, IGF-IR amplification was observed in 14% of tumors [75]. Indeed, Behjati et al. reported no difference in somatic alterations when comparing pediatric and adult osteosarcomas, with chromothripsis amplification occurring at all ages, indicating an age-independence of somatic mutations. These data may facilitate patient selection in future trials examining IGF1R inhibitors as therapeutic agents in osteosarcoma [75].

The current clinical challenge is to prevent recurrences and offer new treatment options for patients with inoperable primary, recurrent disease, and/or metastases. The latter is also well known to be associated with radio and chemoresistance in cancer [233,234].

Although failures have been registered in creating novel targeted therapeutics aiming at the IGF pathway, new agents’ development should continue, evaluating combinatorial strategies for enhancing antitumor responses and better classifying the patients that could best benefit from these therapies [235]. Importantly, combinations are suggested to attenuate possible associated adverse side effects by decreasing therapy dosage, highly significant for pediatric patients. Thus, the utilization of quantitative phosphoproteomics demonstrated the beneficial disease-specific synergistic effect of simultaneous application of pan-tyrosine kinase PKC412 inhibitor and IGF1R inhibitors. The effect of the drug synergy between these inhibitors is different from the simple sum of the single-agent effects at lower than the single-agent dosage [236].

A plausible approach for developing a combinatorial strategy is to focus on the tumor microenvironment (TME) and processes executed therein. Reprogramming the matrix component of the TME could enhance the antitumor effects of available therapies. Matrix mediators’ effects on autophagy and apoptosis could also be recruited in an ongoing effort to harness these processes for new therapeutic strategies [237,238]. Therefore, as new agents are continuously developing, such as anti-tumor-specific antibodies or even specific CAR-T cells, matrix-derived approaches can be part of the combinatorial strategies that enhance antitumor responses.

## 9. Conclusions

The IGF axis is a complex multifactorial molecular system involved in malignancy, and targeting this axis should take into account its level of expression, activation state, accessibility, and functionality of all other interacting components, including all the immune-related events. Significantly, the IGF system directly contributes to tumor growth and, even more notably, to acquired resistance to conventional/focused drugs. Moreover, the IGF axis modulates the expression of ECM components and regulates the cellular TME compartment’s functions. On the other hand, the ECM components modulate the IGF-signaling to facilitate sarcoma progression. Targeting the specific components of the TME and/or reprogramming the TME cell functions in combination with the blocking of the IGF-axis could enhance the exploitation of the respective therapeutic strategy.

## Figures and Tables

**Table 1:** Summary of the IGF-signaling effects on ECM molecules’ expression/activity.

Regulator	ECM Target	Effect	Cells	Ref.
IGF-1 and IGF-2	Aggrecan	Maintaining high expression	Chondrocytes/Chondrosarcomas (In vitro; cell lines)	[125]
IGF-1	PGs and p21	Increased expression	Chondrosarcomas (In vitro; cell lines)	[128]
IGF-1	Xylosyltransferase I and alkaline phosphatase	Increased expression	Osteosarcomas (In vitro; cell lines)	[135]
IGF-1	Collagen I	Increased expression	Osteosarcomas (In vitro; cell lines)	[141]
IGFBP-4	Collagen I	Decreased expression	Osteosarcomas (In vitro; cell lines	[141]
IGF-I	Cysteine protease	Decreased activity	Osteosarcomas (In vitro; cell lines)	[142]
IGF-1 and/or IGFBP-5	Collagen I	Enhance Estrogen-mediated PTH-dependent expression	Osteosarcomas (In vitro; cell lines)	[143]
IGF-1	Collagen II	Increased expression	Chondrocytes (In vitro; rat primary cell cultures)	[147]
IGFBP-1	VCAM-1	Increased expression	Osteosarcoma (In vitro; primary cell cultures; tissue biopsies)	[153]
IGF-1	α5β1-integrin	Increased expression	Chondrosarcoma (In vitro; primary cell cultures)	[156]
IGFBP-3	MMP-9	Decreased activity	Ewing sarcoma (In vitro; primary cell and cell line cultures)	[157]
IGF-IR	MMP-2 and MMP-9	Increased expression	Osteosarcomas (In vitro; cell lines; tissue biopsies)	[158]

**Table 2:** Matrix mediators affect IGF/IGF-IR-dependent sarcoma cell functions.

Regulator	Activity	Effect	Tumor	Ref.
Heparin/HSPGs	Regulate IGF-1/IGF-2- binding to IGFBP-2	Attenuation of IGF signaling/Inhibition of apoptosis	Osteosarcoma cells/Osteoblasts	[160,161]
NG2/CSPG4	Decreases IGFBP3 expression and facilitates IGF-signaling	Decreased tumor size	Murine and human sarcoma models	[164]
Syndecan 2	Co-receptor for IGF-1 and linker to ezrin	Facilitates IGF-I-dependent fibrosarcoma cell migration	Fibrosarcoma	[168]
PAPP-A	Cleavage of inhibitory IGF-binding proteins	Increased free IGF-I, cell growth and downstream IGF signaling	Ewing sarcoma	[179]

**Table 3:** Anti-IGF-IR mAb targeting the IGF axis in sarcomas and their clinical phase of development.

Tumor Type	Drug	Phase	Clinical Results	Safety Results	Ref.
EWS and other solid tumors	Cixutumumab	I/II (only pediatric patients)	Limited activity in EWS	Well tolerated	[221]
EWS and desmoplastic small round cell tumors	Ganitumab	II	Limited activity in EWS	Generally well tolerated	[226]
EWS and other sarcomas	figitumumab	I	EWS objective responses: complete response, partial response, and stable disease in EWS, synovial sarcoma, and fibrosarcoma, lasting over 4months	Well tolerated; mild-to-moderate adverse effects	[223]
EWS	figitumumab	I/II	Modest activity as single agent in advanced E	Good tolerability	[224]
EWS	R1507	II	Partial response	Well tolerated	[225]
Bone and soft-tissue sarcomas	R1507	II	Limited efficacy; overall response rate 2.5%)	Well tolerated	[228]
Resectable osteosarcoma metastases (Group 1), unresectable osteosarcoma metastases (Group 2), and Ewing sarcoma metastases(Group 3)	Robatumumab	II	Limited efficacy in osteosarcoma patients, 6 of EWS patientshave remained healthy after receiving 25–115 doses of robatumumab with remissions of >4 years duration	Well tolerated	[227]

## Data Availability

Data sharing not applicable. No new data were created or analyzed in this study. Data sharing is not applicable to this article.
